# Clinical experiences with 6-monthly paliperidone palmitate beyond the diagnosis of schizophrenia. A retrospective study

**DOI:** 10.1192/j.eurpsy.2023.1045

**Published:** 2023-07-19

**Authors:** S. Benavente López, S. Bolaño Mendoza, A. Parra González, A. Lara Fernández, A. Herencias Nevado, E. Baca García

**Affiliations:** Psychiatry, Hospital Universitario Infanta Elena, Madrid, Spain

## Abstract

**Introduction:**

Long-acting injectable antipsychotics (LAIA) are used in diagnoses other than schizophrenia. Over the last two decades, LAIAs have been developed with less administration frequency, going from 2-weekly presentations to 6-monthly presentations. The 6-monthly paliperidone palmitate has recently been released, allowing a reduction in the frequency of administration compared to the 1-monthly presentation and the 3-monthly presentation. Descriptive studies based on real clinical evidence can be very useful to assess clinical outcomes.

**Objectives:**

The main objective of the study is to describe the use of 6-monthly paliperidone palmitate in patients with schzophrenia, providing variables that objectify the evolution such as the number of psychotic decompensations.

**Methods:**

Retrospective descriptive study with a sample selected by non-probabilistic consecutive sampling, retrospective type, in a time interval of 10 month (n=80). The patients selected were all those who received 6-monthly paliperidone palmitate treatment from after 10 months of use at Hospital Universitario Infanta Elena. A descriptive analysis was performed. Mean and standard deviation were calculated for quantitative variables and N and percentage for categorical variables.

**Results:**

A total of 80 administrations of 6-monthly paliperidone palmitate were performed in the study. None of the patients presented adverse reactions related to the administration of the drug, not reporting local pain or inflammation of the puncture area, except for the characteristic discomfort of an intramuscular puncture. Regarding the efficacy of 6-monthly paliperidone palmitate, none of the patients presented a psychotic decompensation after its administration, maintaining psychopathological stability after the change. The switch to 6-monthly paliperidone palmitate was made from both 1-monthly paliperidone palmitate and 3-monthly paliperidone palmitate, both showing the same efficacy. Regarding tolerability, all the patients who were administered 6-monthly paliperidone palmitate were previously treated with the monthly and quarterly presentation of the same molecule, having presented good tolerability to it, maintaining said tolerability after treatment. change to 6-monthly paliperidone palmitate, with no adverse reaction being recorded after the change. The adherence presented by the patients was very good, performing 100% of the administrations.

**Conclusions:**

6-monthly paliperidone palmitate may be an effective and well-tolerated treatment for the treatment of schizophrenia and other diagnoses such as bipolar disorder or borderline personality disorder. According to objective data, 6-monthly paliperidone palmitate could be an effective and well-tolerated treatment as an alternative to monthly and quarterly presentations of the same molecule. Longitudinal studies must be carried out to confirm this hypothesis.

**Disclosure of Interest:**

None Declared

